# HBV Life Cycle: Entry and Morphogenesis

**DOI:** 10.3390/v1020185

**Published:** 2009-09-01

**Authors:** Stephanie Schädler, Eberhard Hildt

**Affiliations:** 1 Molecular Medical Virology, Institute of Infection Medicine, University of Kiel, D-24105 Kiel, Germany; E-Mail: schaedler@infmed.uni-kiel.de; 2 Faculty of Biology, University of Freiburg, Schaenzlestrasse 1, D-79104 Freiburg, Germany

**Keywords:** hepatitis B virus, entry, morphogenesis, liver disease

## Abstract

Hepatitis B virus (HBV) is a major cause of liver disease. HBV primarily infects hepatocytes by a still poorly understood mechanism. After an endocytotic process, the nucleocapsids are released into the cytoplasm and the relaxed circular rcDNA genome is transported towards the nucleus where it is converted into covalently closed circular cccDNA. Replication of the viral genome occurs via an RNA pregenome (pgRNA) that binds to HBV polymerase (P). P initiates pgRNA encapsidation and reverse transcription inside the capsid. Matured, rcDNA containing nucleocapsids can re-deliver the RC-DNA to the nucleus, or be secreted via interaction with the envelope proteins as progeny virions.

## 1. Introduction

The human hepatitis B virus belongs to the family of *hepadnaviridae*. The *hepadnaviridae* are subdivided into mammalian and avian hepadnaviruses. The mammalian hepadnaviruses include human hepatitis B virus (HBV), woodchuck hepatitis virus (WHV) and the ground squirrel hepatitis B virus (GSHV). The duck hepatitis B virus (DHBV) and the heron hepatitis B virus (HHBV) belong to the avian hepadnaviruses [1]. The *hepadnaviridae* share the following features:

- A partially double-stranded genomic DNA comprising a complete coding strand (negative strand) and an incomplete non-coding strand (positive strand);
- A RNA-dependent DNA polymerase;
- Replication through a pre-genomic RNA template;
- A high degree of species and tissue specificity.

**Figure 1.**
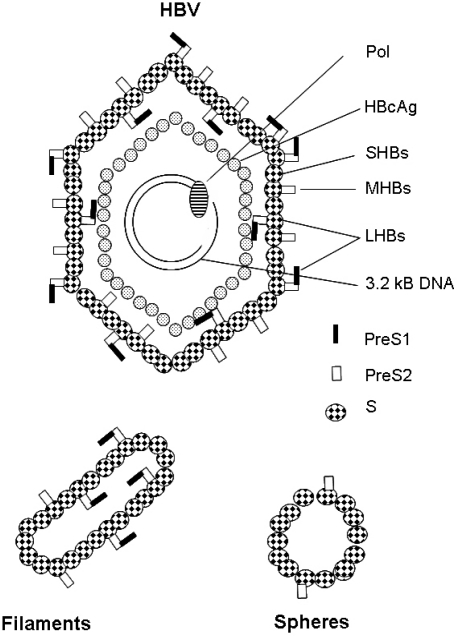
Schematic structure of the HBV particle and subviral particles. HBV is an enveloped virus with a diameter of about 42 nm (42 nm particle). The envelope is formed by the three viral surface proteins LHBs, MHBs and SHBs that surround the viral nucleocapsid. The core protein (HBcAg) forms the nucleocapsid that harbors the partially double-stranded circular DNA genome that is covalently linked to the viral polymerase. In the serum of HBV-positive patients, large amounts of non-infectious subviral particles in the form of filaments or spheres (20nm particles) are found; these are composed of the viral surface proteins, but lack the viral nucleic acid.

The partially double-stranded DNA genome of HBV is about 3.2 kb in size. The viral genome uses all three reading frames and contains at least four different open reading frames, coding for the viral polymerase, the HBc and HBe antigen, the regulatory protein HBx and the preS/S gene encoding the three surface antigens (LHBs, MHBs and SHBs) (Figure 1).

Infection by hepatitis B virus (HBV) can cause an acute or chronic inflammation of the liver. In addition, HBV is a major causative agent for the development of hepatocellular carcinoma (HCC) [2,3]. In the light of this, many studies have focused on the identification of potential viral oncogenic products. Apart from this, many aspects of the viral life cycle are still enigmatic. This review will give an overview of the viral life cycle with the focus on virus binding, entry and morphogenesis of *de novo* synthesized viral particles.

## 2. The structural proteins of HBV

### 2.1. The envelope proteins

**Figure 2.**
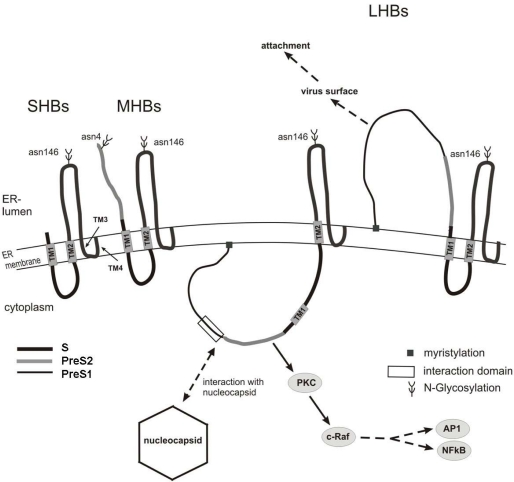
Schematic structure of the HBV surface proteins. The S-domain is common to all three HBV surface proteins. In the case of the large surface protein (LHBs), TM1 is not used as a start transfer signal, resulting in a cytoplasmic orientation of the PreS1PreS2 region. In a fraction of LHBs the PreSPreS2 domain is posttranslationally translocated across the ER membrane. In this case, the PreS1PreS2 domain faces the lumen of the ER. The two forms of LHBs fulfill different functions. This fraction that faces the ER lumen is exposed to the viral surface in the mature viral particle and is involved in the attachment process. The cytoplasmic form mediates the contact to the nucleocapsid and triggers intracellular signal transduction cascades by the interaction of the PreS2 domain with protein kinase C (PKC).

The HBV surface proteins are encoded by one open reading frame that is divided by three in-frame AUG start codons into the following domains: PreS1, PreS2 and S. The large HBV surface protein (LHBs) encompasses the PreS1 domain (108 or 119 aa depending on the genotype), the PreS2 domain (55aa) and the S domain (226 aa); the middle surface protein (MHBs) encompasses the PreS2 and S domain and the small (SHBs) consists of the S domain [4] .The S-domain, that is common to all three surface proteins, harbors at Asn-146 an N-glycosylation site, which is partially used in all three surface proteins [1,5,6]. Moreover, there is in the PreS2 domain at Asn-4 a glycosylation site that is used in MHBs, but not in LHBs (Figure 2). In addition, there is evidence that the Pre-S2 domain of MHBs and, to a minor extent, of LHBs from HBV genotype C and D is partially O-glycosylated at Thr-37. Genotype A, containing no Thr at position 37 or 38, is not O-glycosylated [7].

HBV envelope proteins are integral membrane proteins that are anchored by the S-domain to the membrane [8,9]. Membrane insertion of the S protein is initiated by an N-terminal signal sequence (aa 8–22) that is not cleaved and forms the first transmembrane region (TM1). In the mature protein, the aa 23–79 face the cytoplasm. At aa position 80–98 a second signal that forms TM2 directs the translocation of the growing chain though the ER membrane into the ER lumen, until at about aa 170 the hydrophobic C-terminus of the S-domain that is localized within the ER membrane starts. The detailed structure of the C-terminal region (aa 170–226) is not fully understood and is considered as one or two transmembrane regions (TM3/4) [9,10]. In the complete S protein, the N-terminus (aa 1–7) and the loop between aa 99 and aa 169 face the lumen of the ER, the domain between aa 23–79 face the cytoplasm. The luminal orientation of the loop between aa 99–169 enables the N-glycosylation of Asn-146 by the N-glycosyltransferases that are localized within the lumen of the ER. Moreover, this loop contains the major conformational epitope of the HBV surface antigen (HBsAg). After budding of the mature viral particle, these luminal domains are exposed on the external surface of the viral particle [11–13].

In the case of the MHBs protein there is no difference in the topology of its S-domain as compared to SHBs. The N-terminal PreS2 domain of MHBs is cotranslationally translocated into the ER-lumen resulting in the accessibility of Asn 4 to the N-glycosyltransferases (Figure 2). Therefore, MHBs is found in three forms: the unglycosylated (p30), the monoglycosylated (gp33) and the biglycosylated (gp36) [5,11].

LHBs shows an unusual biosynthesis. Its hydrophilic PreS1-PreS2 domain is not cotranslationally translocated, since TM1 of the S-domain is not used as a cotranslational signal sequence. As a consequence, the PreS1-PreS2 domain and a part of the S domain, up aa 79, remain on the cytosolic face of the ER. TM2 anchors the growing LHBs in the membrane and causes translocation of the downstream sequences into the ER lumen, enabling glycosylation at Asn-146 of the S-domain that still faces the ER-lumen [14–16]. Due to this topology, the Asn-4 in the PreS2 domain is, in contrast to MHBs, not accessible to N-glycosylation [14,17]. The glycine residue at aa 2 of the PreS1 domain in LHBs is myristylated [18,19]. An interesting aspect is that in about 50% of the LHBs proteins a posttranslational translocation [20] occurs, resulting in a dual membrane topology of the mature LHBs. As a consequence of this posttranslational translocation, the “unused” TM1 is integrated into the ER membrane and in this fraction the PreS1-PreS2 domain faces the ER lumen. There seems to be an equilibrium between the two topologies of LHBs, and recent reports suggested that chaperons are involved in the control of the posttranslational topological reorientation [21–23]. With respect to the viral life cycle, the different topological forms of LHBs have different functions. In the mature secreted virion, the PreS1-PreS2 domain originally oriented to the ER lumen is exposed on the outer surface of the viral particle and is important for the virus-cell interaction (Figure 1). The form of the LHBs protein that faces the cytoplasm with its PreS1-PreS2 domain plays an essential role in virus morphogenesis by interacting with the nucleocapsid [24].

Apart from these morphogenic functions, the cytoplasmic orientation of the PreS2 domain is associated with the additional function of PreS2 as regulatory protein. Cytoplasmic PreS2 is found in LHBs and in C-terminally truncated MHBs proteins [2,17,25–27] that are encoded by 3′-end truncated pres/S sequences isolated from HBV-associated HCCs. In this conformation, the PreS2 domain binds to and activates PKC. The PreS2-dependent activation of PKC results in the activation of the c-Raf/MEK signaling cascade that controls the expression of a variety of cellular and viral promoters [28]. PreS2-dependent activation and HBx-dependent activation share a variety of common features [2,29]. It has been shown that functionality of HBx-or PreS2-dependent activation is crucial for HBV replication. Regarding HBV expression, the selective knock-out of PreS2 or HBx can be compensated by the respective other unaffected HBV regulatory protein [29,30]. However, simultaneous knock-out of both PreS2 and HBx abolishes HBV replication [29].

The HBV surface proteins are not only part of the viral particle. They are suggested to bud from the post-ER pre-Golgi membranes [31] without envelopment of nucleocapsids into the lumen of vesicular structures and are finally released by secretion. These subviral particles have a diameter of 20 nm and an octahedral symmetry [32]. In addition, there are filaments of variable length (Figure 1). As compared to the virion, the subviral particles are highly overproduced. In the serum of HBV-infected patients, a 10,000-fold excess of subviral to viral particles can be found. The relevance of the subviral particles for the viral life cycle is not understood. These particles may interfere with the host immune system or support the infection process [33].

A detailed analysis revealed that the HBV subviral particles form by self-assembly of the S protein into branched filaments in the lumen of the ER. These long filaments are then folded and bridged for packing into crystal-like structures before they are transported by ER-derived vesicles to the ER-Golgi intermediate compartment (ERGIC). In the ERGIC, they are unpacked and relaxed. Due to their size, further progression through the secretory pathway might be limited. Therefore, their conversion into spherical particles is required. Small branched filaments can be formed by the L protein in the ER lumen, but these filaments are not packed into transport vesicles, accounting for the retention of the L protein within cells [34].

### 2.2. The nucleocapsid

The HBV nucleocapsid is formed by the core protein (HBcAg) that is conserved between the different genotypes [35]. HBcAg encompasses 183 or 185 aa depending on the genotype, and the primary sequence of HBcAg can be divided into two parts: 1) The N-terminal 149 or 151 aa (depending on the genotype) are sufficient for the self-assembly of capsids. This part of the HBcAg is called the assembly domain. 2) The C-terminal 34 aa, designated the protamine domain, is rich in arginine residues that confer a positive charge to this domain. This domain is essential for the packaging of the pregenome / HBVPol complex [36,37].

HBcAg can be overproduced in pro- and eukaryotic expression systems and assembles in these systems to capsid particles [38]. Capsid assembly starts with the formation of HBcAg dimers that are crosslinked by a disulfide bridge between Cys-61 [39–41]. *In vitro* capsid assembly can proceed independent of cellular factors [42,43]. Crucial factors controlling assembly of purified HBcAg dimers are the concentration of HBcAg dimers and the buffer composition [42]. During the viral life cycle, capsid formation initiates with the binding of the Pol-complexed viral pregenome, but depends on cellular factors as chaperones and kinases [44–49]. Moreover, there are reports describing the incorporation of PKC in the HBV capsid [50]. A recent report established a correlation between PKC-dependent phosphorylation of the assembly domain at Ser-106 and an increased level of assembled core. Moreover, in addition to the promotion of capsid assembly, the phosphorylation at Ser-106 seems to increase the stability of the assembled core, although the ratio of α-helical content was decreased in the capsid [51]. These factors might contribute to decrease the threshold concentration that is required for the initiation of capsid formation.

In principle, HBcAg dimers can assemble into two different types of particles: on the one hand, particles with a diameter of 30 nm that consist of 90 HBcAg dimers and display a T= 3 symmetry, and on the other hand larger particles with a diameter of 34 nm, that are assembled by 120 dimers and display a T = 4 symmetry [52]. Although both particle forms can be found in HBcAg-producing systems, in infectious viral particles mainly the T = 4 capsids are found. However, in 10% of the virions T=3 capsids are found [12]. Based on cryo-electron microscopy [53,54] and crystallization of the capsid [43,55] details of the capsid structure were revealed. The most prominent features are surface spikes, flanked at either side by holes. Each spike can be arranged in one of two non-identical environments that are either part of two hexagons or of a hexagon and a pentagon. The spikes are formed by the core dimers. From each subunit, two anti-parallel α-helices, which are connected by a short loop in between aa 78–83, associate into a four-helix bundle [43,53–55]. The loop connecting the α-helices forms the spike tip and represents the major epitope of the capsid antigen. The assembled capsid is neither a tight shell nor a rigid inflexible particle. The conversion of the RNA pregenome into the DNA genome requires the import of nucleotides into the capsid lumen. The capsid shell contains pores with a diameter between 12 Å – 15 Å that enable the diffusion of small molecules into and out of the capsid lumen [56–58]. Based on the observation that foreign sequences can be inserted into the spike tip in between aa 78–81 without affecting the capacity to form capsids [59,60], it could be shown that the capsid structure is both highly stable and enormously flexible [56,61]. Induction of a conformational change from the outside is associated with a change in the internal capsid organization [56]. This conformational cross talk between capsid lumen and surface might be relevant for the control of capsid maturation during conversion of the RNA pregenome into the mature DNA genome [57,58,62].

Affecting nucleocapsid formation could be an alternative therapeutic approach to control HBV infection. The heteraryldihydropyrimidine (HAP) Bay 41-4109 recently was found to act in an HBcAg-specific manner and thereby inhibits virus production [63]. A more detailed analysis revealed that Bay 41-4109 can accelerate and misdirect capsid assembly [64]. Depending on the ratio of inhibitor molecule to HBcAg dimer, Bay 41-4109 can exert a stabilizing effect (up to a ratio of one inhibitor molecule per two dimers) or a destabilizing effect yielding large non-capsid HBcAg aggregates (Bay41-4109: dimer ratio of 1:1 or greater) [64–66]. From these data it is concluded that Bay41-4109 affects virus replication at low concentrations by induction of an inappropriate assembly of the capsid, and at higher concentrations by misdirecting the assembly from capsid formation to the formation of large HBcAg aggregates.

## 3. The infection process

### 3.1. Attachment

According to a general concept of viral infection, the first step is an energy-independent attachment of the viral particle to a structure at the host cell surface. After the primary attachment, which is characterized by low affinity and reversibility, the virus particle is transferred to a more specific receptor. In case of enveloped viruses the binding to the receptor normally is followed by a fusion step, either at the plasma membrane or in an endosomal compartment [67]. Many aspects of the initial steps in the HBV life cycle at the present are still enigmatic. However, based on the establishment of different *in vitro* infection systems significant progress has been achieved recently.

### 3.2. Experimental systems

Primary human hepatocytes (PHH) are a classic *in vitro* infection system to study HBV infection [68,69]. The major disadvantage of these cells is their limited availability and their heterogeneous quality, varying from donor to donor. Moreover, there is a low infection efficiency leading to only few percent of infected cells [70,71].

Primary hepatocytes from *Tupaia belangeri* (PTH) represent an alternative cell culture system [72]. PTHs are more readily available and there is less variability between different preparations. The infectability of PTHs is comparable to that of PHHs. However, in contrast to PHH, PTH can be infected by woolly monkey HBV in addition to HBV [73,74]. A drawback of using PTHs as an infection model, however, is that this cell culture system is less characterized and the cross-reactivity of many antisera specific for mouse or human targets with the corresponding *Tupaia* protein so far is not clear.

Based on this, the availability of a human hepatoma cell line that can be infected with HBV is desirable. For the human hepatoma cell line HepG2 there are many reports describing a specific binding and uptake of HBV [75–78], however, there are only two reports of infection of HepG2 that were cultivated in the presence of DMSO and 5-aza-2`deoxycytidine [76,79]. A new hepatoma cell line, HepaRG, established from a female HCV-positive patient with an HCC was reported to be susceptible to HBV infection after differentiation in DMSO and hydrocortisone [80] and to enable reproducible infection by HBV.

A general problem in the analysis of productive infection is to differentiate between input and *de novo* synthesized viral or subviral particles. An unequivocal marker for productive HBV infection is the formation of covalently closed circular (ccc) DNA (for a recent review see [81]) that can be detected by Southern blotting or by a real-time PCR approach for selective amplification of cccDNA, but not of the other viral DNAs [82]. The cccDNA amplification can be detected for infected PTH and PHH, but in the case of HepaRG cells there seems to be no amplification of cccDNA [83]. Detection of the viral mRNAs provides an additional approach to discriminate between input and *de novo* synthesis.

When using enriched or purified viral inocula, detection of HBeAg by ELISA represents a good marker, since after the enrichment procedure HBeAg is not present in the inoculum. HBsAg detection is more sensitive as compared to HBeAg, it requires, however, multiple cell washing steps [84].

### 3.3. Virus-cell interaction

HBV infection is thought to follow a multistep process. While for the DHBV system heparin or dextran sulphate have no effect on the infection process [85], in the case of HBV there are reports about the relevance of the initial attachment to the carbohydrate side chains of hepatocyte-associated heparan sulphate proteoglycans as attachment receptors for HBV infection [86,87]. This interaction is suggested to initiate the multistep entry process of HBV and is followed by yet unknown high-affinity step(s) mediating HBV uptake. Identification of the “HBV receptor” or of HBV binding partners is one of the challenging open questions in the field of HBV biology. There is a constantly growing list of proteins that were found to bind to HBV, but for none of these potential binding factors is there convincing evidence of its essential relevance for the infection process (for a detailed list see [88]). While the cellular structures that mediate viral binding and entry are less understood, more is known about the viral structures involved in binding and entry. A milestone in the characterization of viral prerequisites for the binding to hepatocytes was the observation of Neurath *et al.* in 1986 [75]. Neurath and colleagues reported that a short fragment of the surface protein encompassing aa 21–47 of the PreS1 domain (this corresponds to aa 10–36 in genotype D, E and G) binds to HepG2 cells and completes the binding of HBV to these cells. Consistent with this finding, it has been observed that aa 3–77 of HBV PreS1 are crucial for infectivity [89]. Paran *et al.* identified a QLDAPF sequence motif (corresponding to aa 18–25) as an essential domain for HBV binding [76]. A further structural prerequisite for the infectivity of HBV is the myristylation at glycine 2 of the PreS1 [18,19]. It was observed that a myristylated peptide PreS1 domain showed significantly stronger binding to HepG2 cell-derived membranes as compared to the non-myristylated PreS1-domain [90]. Detailed studies revealed that acylated peptides encompassing the N-terminal part of the PreS1 domain efficiently inhibit HBV and HDV infection [91–94]. One essential parameter is the hydrophobicity of the N-terminal acyl residue. The increase in the inhibitory potential is associated with an increase in the chain length of the fatty acid. A pentanoyl group is less efficient than a decyl group, which is less efficient than a myristoyl group (C14). Regarding the peptide sequence, it was revealed that residues 1–8 and 19–28 are dispensable for the inhibitory effect; residues aa 9–18, however, are crucial. In accordance with this, recombinant HBVs mutated between aa 9–18 of the PreS1 domain are not infectious [91].

The mechanism by which these peptides exert their inhibitory effect is presently unclear. The very low IC50 of 8 nM and the observation that these peptides are inhibitory even if they are added after the virus attachment has occurred argue against a simple competitive inhibition. Moreover, there is no clear correlation between the specificity of peptide binding, on the one hand, and susceptibility of the respective cell for HBV infection on the other hand. Analysis of the bio-distribution of these peptides in immunodeficient urokinase-type plasminogen activator (uPA) mice, repopulated with primary human or *Tupaia belangeri* hepatocytes, demonstrates an accumulation of the acetylated peptides in the liver, but no preferential binding to the implanted PTHs or PHHs [95]. Based on this, it can be speculated that these peptides inhibit viral infection by interfering with signal transduction cascades that regulate HBV infection or with early post-entry steps.

Although the PreS1 domain contains the major cell attachment epitope, there are reports about further epitopes outside the PreS1 domain that are involved in HBV-cell attachment. Paran *et al.* describe the existence of a secondary attachment site in the S domain [76]. Moreover, antibodies recognizing epitopes within the PreS2 domain or the S domain were found to inhibit infection [96]. However, it is unclear whether these antibodies act by directly masking an essential sequence for HBV-cell attachment, or whether their binding acts as a spacer, preventing the close contact between the virus and the cell surface. Moreover, the interference with a post-entry step is possible.

Data from the DHBV system suggest that *hepadnaviridae* are internalized by an endocytotic step [97–99]. It was observed that DHBV particles colocalize with fluorophor-labelled transferrin in the endosomal compartment [100]. Moreover, it has been demonstrated that bafilomycin A1, which inhibits vacuolar proton ATPases, impairs infection.

### 3.4. Entry and release of the nucleocapsid into the cytoplasm

Productive infection with HBV requires delivery of the genome into the nucleus [81]. Resulting questions concern the entry of the virus/nucleocapsid into the cell and the subsequent transport of the genome towards the nucleus. In contrast to viruses harboring type I fusion proteins on their surface, HBV does not possess a classic fusion peptide sequence [67,101,102]. In a recent report, a fusogenic function was ascribed to the PreS1 domain of HBV [103]. Based on sequence analysis, it was suggested by Rodriguez-Crespo *et al.* that the N-terminus of the S-domain (aa 1–23), including the first transmembrane region (TM1), might act as a fusogenic sequence [104]. This hypothesis is supported by the observation that a chimeric fusion protein of influenza virus, hemagglutinin, with the sequence aa 7–18 of the S domain, showed significant hemifusion activity [101]. Moreover, it was demonstrated that DHBV subviral particles upon low pH treatment expose hydrophobic domains on their surface that could mediate membrane contact [105]. Further analysis revealed that a decrease in the hydrophobicity of the TM-1 domain in DHBV L protein but not in S-protein resulted in a loss of infectivity. Moreover, *in vitro* experiments with synthetic peptides corresponding to TM1 indicate that the hydrophobicity of TM1 is required for aggregation and lipid mixing of phospholipid vesicles [100,105]. Although these data suggest that TM1 could act as a fusogenic sequence, so far there is no direct experimental evidence for fusion to host cell membranes during HBV entry.

In the case of genotype ayw, the PreS2 domain of HBV harbors between aa 41–52 a membrane-permeable peptide designated TLM (translocation motif). The presence of this TLM is conserved in all *hepadnaviridae* [106]. The TLM belongs to the family of membrane-permeable peptides. Fusion of the TLM to other peptides or proteins enables their energy and receptor-independent translocation across cellular membranes into the cytoplasm [30,106–109]. The functionality of the TLM as a membrane-permeable peptide depends on a defined pattern of hydrophilic and hydrophobic amino acids that form a labile amphipathic alpha helix [17,106]. Fusion of the TLM to HBcAg revealed that fully assembled nucleocapsids that are decorated on their surface with TLM-peptides are able to translocate across cellular membranes and deliver the packaged nucleic acid to the nucleus [108]. These data demonstrate that even particles, if they bear TLM peptides on their surface, are able to translocate across cellular membranes. Based on this, it was analyzed whether the TLM peptide could play a role in the HBV entry process using the DHBV system. In contrast to HBV, DHBV harbors two TLMs in the PreS domain. Infection experiments revealed that destruction of the TLMs, or even of one TLM, abolished infectivity [99]. More detailed analysis revealed that TLM-deficient DHBV particles still bind to the cell and are able to enter the endocytotic pathway, but the TLM deficient mutants accumulate in an endosomal compartment. Further experiments revealed that in the endosomal compartment a proteolytic processing of the internalized viral particle occurs, resulting in an unmasking of the TLM peptide. It was concluded that, due to the endosomal proteolytic processing, unmasked TLM enabled the translocation across the endosomal membrane into the cytoplasm, where the proteolytically processed envelope dissociates from the nucleocapsid. The capacity of viral particles that have been proteolytically processed by preincubation with endosomal lysate to translocate across cellular membranes has been shown for HBV and DHBV. Incubation of HepG2 cells with proteolytically processed HBV or LMH resulted in a productive infection of these cells, which are not permissive to infection with the unprocessed virus [99].

Based on these data, it was suggested that *hepadnaviridae* could deliver their nucleocapsid into the cytoplasm not by a fusion process, but by a novel mechanism that is based on membrane translocation [99]. However, there are publications arguing against this model: HDV entry does not depend on the functionality of a TLM [110,111], and mutation or deletion of the TLM in HBV seems not to affect its infectivity [110,112]. Recent data, however, raise the question whether HDV is a suitable model system to study HBV entry. While chimeric particles harboring woodchuck envelope proteins are unable to infect PHHs, a recombinant HDV assembled with envelope proteins of WHV infects PHHs, indicating significant differences between the entry process of HDV and HBV [113]. Detailed analysis of the TLM-mutated HBV [112] reveals that due to the partial deletion of either the C-terminal or N-terminal part of the TLM a novel functional TLM was generated. However, destruction of the TLM by point mutations that convert the structure to a stable beta sheet resulted in a complete loss of infectivity using PHH (E. Hildt’s unpublished results).

### 3.5. Import of the genome into the nucleus

Lipofection of mature nucleocapsid [114] or transfection of primary human hepatocytes or hepatoma cells with membrane-permeable nucleocapsids [108] indicates that the nucleocapsid moves by a directed transport towards the nucleus. This can be deduced from the kinetics of intracellular trafficking [115]. A perinuclear accumulation after delivery of the nucleocapsid into the cytoplasm can be observed within 15 min, while a diffusion-based process would take over 1h [116]. A central role for the intracellular trafficking of the nucleocapsids is ascribed to the microtubule system [114]. The controversial issue of the relevance of actin filaments for intracellular nucleocapsid transport has been discussed [108,117].

Productive viral infection requires the transport of the HBV genome into the nucleus, where the conversion into cccDNA occurs (recently reviewed in [81]). It is questionable whether the import of the viral genome into the nucleus occurs in association with HBcAg or not. The limited efficiency of the available infection systems, as well as the very small amounts of nucleocapsids which are finally released into the cytoplasm, make it difficult to address this question. One approach to investigate this is based on digitonin-permeabilized cells, which are subsequently exposed to nucleocapsids [118]. Based on this experimental system, a phosphorylation-dependent binding of the core particle to the nuclear pore complex was observed [119]. According to their previous observation that PKC can be encapsidated into the core particle [50], the authors assume that the encapsidated PKC phosphorylates C-terminal Ser residues in the core protein giving rise to mature phosphorylated progeny core particles. However, this appears to be in contrast to more recent observations that correlate HBV capsid maturation with stepwise dephosphorylation [58,62].

Further work of this group suggested that immature capsids reached the basket of the nuclear pore complex, but neither released capsid proteins nor immature genomes into the nucleoplasm. In the case of mature capsids, intranuclear staining for HBcAg was observed [120]. However, the digitonin permeabilization procedure affects the integrity of the cell. The permeabilization kills the cell and causes loss of cellular proteins. Traces of digitonin might affect the stability of the nucleocapsids. Moreover, the chosen antiserum (Dako HBcAg) detects HBcAg dimers as well as fully assembled particles, and, therefore, does not allow the conclusion that assembled particles have translocated into the nucleus. Electron microscopy data after microinjection of nucleocapsids into *Xenopus laevis* oocytes, however, demonstrate that in this system the capsid passed the nuclear pore and entered the nuclear basket. However, even immature nucleocapsids were found to enter the nuclear basket [121]. It is assumed that only disaggregation of the mature nucleocapsid can occur, resulting in the release of the polymerase-linked viral genome into the nuclear basket.

In a recent report, an efficient system for gene transfer into hepatocytes based on cell-permeable nucleocapsids was described [108]. The cell permeability of the nucleocapsids was achieved by fusion of the TLM peptide [106] to the HBcAg. Dimers of TLM-HBcAg assemble into the icosahedral capsid. This peptide enables the receptor-independent translocation of cargo (proteins or peptides that are fused to the TLM) across the plasma membrane without affecting the integrity of the cell [30,107,122]. These TLM-nucleocapsids translocate as fully assembled particles across the plasma membrane without affecting the cellular integrity. Finally, the HBV genome, or its derivative, packaged into these TLM nucleocapsids, is efficiently expressed, indicating that a productive trafficking ending with expression of the packaged genome occurs [108]. Using this system and an antibody that selectively recognizes fully assembled nucleocapsids (mab 3120) [123], no evidence for nuclear import of nucleocapsids was obtained, but a perinuclear accumulation could be observed. If the disassembly of the nucleocapsid does not occur within the nucleus, the viral genome that is linked to the polymerase, and therefore is too big for a free diffusion though the nuclear pore complex, must be transported actively into the nucleus. One possibility could be the association to HBcAg dimers that possesses a nucleic acid binding domain and an NLS sequence [53]. Another possibility could be that the polymerase mediates the final import of the viral genome. This is supported by the observation that the Pol-DNA complex is efficiently imported into the nucleus. Deproteinization of the viral genome, however, caused retention in the cytoplasm [118]. Recently it was revealed that the TP-domain of HBV polymerase harbors a functional bipartite nuclear localization signal that is crucial for the HBV infectivity [124].

### 3.6. Replication

#### 3.6.1. rcDNA to cccDNA conversion

At the end of the viral entry process, the viral genome is delivered into the nucleus. The viral genome exists at this stage as rcDNA (relaxed circular DNA). rcDNA consists of a complete (−)-DNA strand covalently linked to the viral polymerase P at its 5′ end, and an incomplete (+)-DNA strand with an RNA oligonucleotide at its 5′ end, which serves as primer for the (+)-strand synthesis. To establish a viral infection, the viral genome has to be present in a stable form within the infected cell. In the case of HBV, the viral rcDNA is converted into a nuclear, episomal covalently closed circular DNA (cccDNA), which represents the central intracellular intermediate in viral replication and also serves as an experimental marker for the successful establishment of an infection [125,126]. For both genome amplification and cccDNA formation, the shorter (+)-DNA strand has to be completed, both strands need to be covalently ligated and the obstructive terminal modifications must be removed. The mechanism of how the viral polymerase and RNA primer is removed from the (−)-DNA strand and the (+)-DNA strand, respectively, is still not fully understood. Two independent studies identified a protein-free rcDNA containing the identical nucleotide sequence as the encapsidated rcDNA, but the polymerase is not bound anymore to the (−)-DNA strand and might be an intermediate during cccDNA formation [127,128]. Infection experiments with primary *Tupaia* hepatocytes showed that blocking reverse transcriptase activity of the viral polymerase strongly reduces cccDNA formation [73,74]. Furthermore, recent *in vitro* experiments revealed that DDX3 DEAD-Box RNA helicase is incorporated into nucleocapsids inhibiting reverse transcription, which further leads to a reduced level of double-linearized DNA (dlDNA) [129]. Taken together, these findings suggest a role of the P protein in completion of the (+)-DNA strand. However, the detailed process of cccDNA generation still remains unclear and needs to be further investigated.

#### 3.6.2. pgRNA transcription from cccDNA

The nuclear cccDNA serves as template for the synthesis of the pregenomic RNA (pgRNA), an RNA intermediate for viral replication and other subgenomic RNAs. The bicistronic pgRNA has two major roles in viral life cycle: it serves as translation and reverse transcription template. The pgRNA is an overlength transcript containing a second copy of the direct repeat 1 (DR1), the ɛ signal and a poly-A tail, serving as a transcript for the translation of the 90 kDa viral polymerase, the 21 kDa core protein, and a 24 kDa precursor early antigen. Second, it serves as template for reverse transcription of the viral (−)-DNA strand and is, therefore, indispensable for viral replication. Besides the pgRNA, there are three additional subgenomic RNAs coding for the surface proteins (2.4 kb RNA and 2.1 kb RNA) and the HBx protein (0.7 kb RNA) [81,130]. Transcription of all hepadnaviral RNAs is processed by host cell polymerase II. Besides the wt RNA, there are splicing variants being translated into hepatitis B splice-generated proteins and encapsidated into defective viral particles [131].

#### 3.6.3. Reverse Transcription

Hepatitis B viruses, as well as other members of the *hepadnaviridae*, use pgRNA as replication intermediate for reverse transcription. First, the pgRNA-polymerase complex is packed in the lumen of assembling capsids, whereas the viral polymerase binds to the encapsidation signal ɛ which is a ciselement on the pgRNA. How the interaction of ɛ and the polymerase takes place is currently not understood in detail, but it is supposed to play a role in the recruitment of core protein homodimers, which finally leads to capsid formation through self-assembly. Besides initiation of pgRNA-polymerase encapsidation, the ɛ-polymerase interaction also induces reverse transcription, whereby first the (−)-DNA strand is synthesized, followed by (+)-DNA strand generation finally leading to rcDNA. A protein-priming mechanism is crucial for the start of DNA synthesis. A short DNA oligonucleotide covalently linked to the polymerase binds to ɛ and initiates (−)-DNA strand synthesis. Besides these two factors, the core protein is also essential for reverse transcription, as concluded from several reports which could clearly demonstrate that deletions or modification of the C-terminal assembly region of the core protein lead to defective DNA synthesis [39,132–134]. The mature secreted HBV virions have completed reverse transcription, which takes place in intact nucleocapsids and contain only DNA. After the DNA genome is synthesized, the nucleocapsid can either continue with the viral life cycle and interact with envelope proteins [135,136] and get secreted as infectious virions [137], or they can re-deliver their rcDNA to the nucleus and build up a cccDNA pool within the nucleus [138].

## 4. Morphogenesis

### 4.1. Capsid maturation

HBV nucleocapsid formation starts when the complex of the RNA pregenome, HBV polymerase and HBcAg dimers has formed [44,47]. When nucleocapsid assembly is completed, the conversion of the RNA into single-stranded and then into partially double-stranded DNA takes place (reviewed in detail in [81]). In contrast to the nucleocapsids isolated from secreted virus, that contain only mature partially double-stranded DNA, intracellular nucleocapsids show all these different stages of the viral DNA synthesis. Based on these observations, it was concluded that the early RNA-containing capsids (the immature nucleocapsid) are not incorporated into viral particles [139]. The resulting questions are whether there are changes in the capsid structure associated with capsid maturation that enable discrimination between the immature and the mature nucleocapsid. For DHBV and HBV, it was reported that mutations in Pol that destroyed the reverse transcriptase activity resulted in an accumulation of immature nucleocapsids that are not enveloped [140,141] Further experiments based on the DHBV system demonstrated that the envelopment of the nucleocapsid occurs at a late stage of the replication cycle [140].

**Figure 3.**
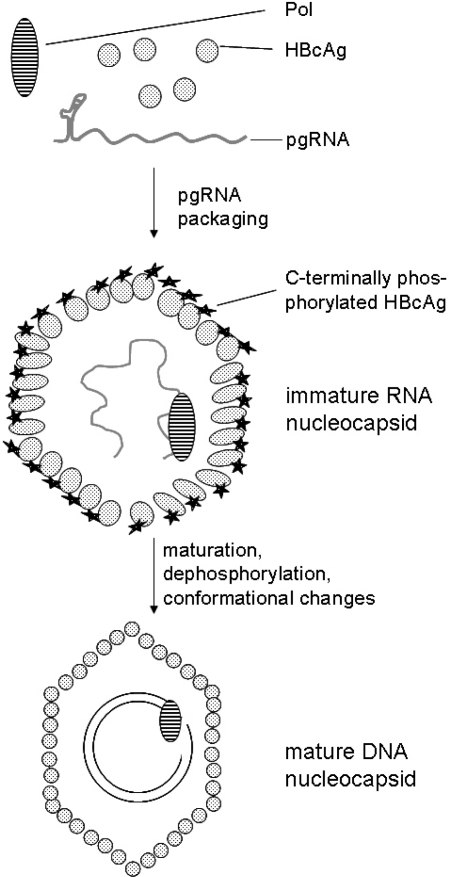
Genome packaging and nucleocapsid maturation. HBV nucleocapsid formation starts when the complex of the RNA pregenome, HBV polymerase and HBcAg dimers has formed. Efficient packaging of the RNA pregenome requires phosphorylation in the C-terminal part of the core protein. Conversion of the immature RNA-containing nucleocapsid to the mature DNA-containing nucleocapsid is associated with dephosphorylation and conformational changes. These significant differences in the structure between the RNA-containing immature nucleocapsid and the mature nucleocapsid trigger the envelopment of the mature nucleocapsid.

Detailed analyses revealed that capsid maturation is associated with a dephosphorylation of the nucleocapsid (Figure 3). Phosphorylation is required for efficient RNA packaging. In the case of HBV, it has been shown that three Ser-Pro-residues (Ser 155, 162, 170) that are located in the C-terminal domain can be phosphorylated [142]. Further analysis based on *in vitro* experiments in HepG2 cells revealed that Ser-162 in the HBV core protein is necessary and sufficient for the encapsidation of HBV RNA. However, both Ser-162 and Ser-170 are required for the production of HBV DNA replicative intermediates. The core Ser-155 is essential for the formation of relaxed circular DNA intermediates. Destruction of these phosphorylation sites by a conversion of Ser to Ala resulted in a nuclear accumulation of these nucleocapsids that do not contain significant amounts of DNA. HBx is proposed to support core phosphorylation at these residues to different extents, and thereby to exert a regulatory effect on HBV replication [62].

The identity of the kinase(s) that are involved in the phosphorylation of these residues is not fully understood. Based on *in vitro* experiments, protein kinase C [119] or members of the SPRK kinase family are suggested to be involved [143].

Analysis of DHBV capsid phosphorylation by detailed mass specrometric analyses revealed that the core protein from immature nucleocapsids was phosphorylated on at least six sites, whereas the mature nucleocapsid was completely dephosphorylated [57]. In accordance to this, it was observed that mutation of the DHBV core phosphorylation sites to Ala completely blocked reverse transcription at a very early stage. Aspartate mutations, however, enabled complete first-strand DNA synthesis, but were defective in accumulating mature double-stranded DNA. This reflects, on the one hand, the instability of the Asp-core mutants, and, on the other hand, the block in the mature second-strand DNA synthesis [144].

Based on the data from the HBV [58] and DHBV [144] systems, it has been concluded that nucleocapsid maturation can be described by a sequential phosphorylation (immature nucleocapsid) and dephosphorylation (mature nucleocapsid) [58]. This dephosphorylation during capsid maturation is associated with significant differences in the structure between the RNA- and the DNA-containing cores (Figure 3). In particular, there is a strong change affecting a hydrophobic pocket close to the spike that is required for the interaction of the preS1 domain with the nucleocapsid [12,145]. This pocket is formed largely by residues that upon mutation have been shown to lead to abnormal viral secretion [146].

### 4.2. Envelopment and budding

In contrast to type C retroviruses or lentiviruses, where mutants with impaired envelope protein formation are still released coated with lipid bilayer, in the case of HBV the envelopment of the mature nucleocapsid strictly depends on the presence of the viral surface proteins. However, it was shown that MHBs is dispensable for virus production [89,147]. Formation of LHBs and SHBs are strictly required [11,148]. Moreover, virion formation requires that in a fraction of LHBs the PreS1PreS2 domain faces the cytoplasm [14]. Fusion of a secretion signal to the N-terminus of LHBs results in the exclusive formation of LHBs molecules that expose the PreS1PreS2 domain to the lumen of the ER. This enables the formation of subviral particles [16], but prevents the secretion of viral particles [24,149]. This observation is supported by findings from the DHBV system [150,151]. Here it was found that the L protein is required for the envelopment of the nucleocapsid. Absence of L results in transport of the capsid to the nucleus, reimport and amplification of the viral genome. For DHBV, aa 116–137 of the PreS domain were considered to be essential for virus morphogenesis [152]; in the case of HBV, aa 103–124 (aa 92–113 depending on the genotype) [24].

It is assumed that this part of the PreS1 domain interacts with the capsid during envelopment. This hypothesis is supported by the observation that mutations within this part of PreS1 impair capsid envelopment. Moreover, *in vitro* binding assays with HBV PreS1-derived peptides and recombinant peptides support this [153] (E. Hildt’s unpublished results). In addition to the PreS1PreS2 domain that faces the cytoplasm, there is a short loop in the S domain between TM1 and TM2 that faces the cytoplasm and could interact with the nucleocapsid [12,13,153,154]. Deletions within this domain inhibited virion formation, while the production of subviral particles was not affected.

To identify nucleocapsid residues that are crucial for envelopment, a variety of natural and engineered mutants were analyzed. Based on these experiments, it was concluded that the spike tip seems to have no impact on the capsid envelopment [146]. In contrast, it was observed that a peptide that binds to the spike tip prevents secretion of mature viral particles [155]. Cryo-electron microscopy of HBV particles purified by sucrose density gradient centrifugation supports the observation that the spike tip interacts via electrostatic interactions with HBsAg [13]. Mutagenesis of HBcAg further demonstrated that the aa residues clustered around the base of the spike and in the grove between the spikes are essential for the interaction of the nucleocapsid with the envelope [146]. Electron microscopy data from CsCl-purified HBV particles support this hypothesis [12], while electron microscopy of sucrose gradient purified viral particles [13] fails to demonstrate stable envelope contacts at these sites.

Mature hepadnaviral nucleocapsids form in the cytoplasm. For DHBV, it has been shown that mature nucleocapsids attach to intracellular membranes. This attachment does not require the presence of envelope proteins. Immature nucleocapsids do not bind [156]. The exact mechanism that mediates the delivery of mature nucleocapsids to the post-ER, pre-Golgi-compartment [31], where envelopment occurs, is presently not understood. For retroviruses and some enveloped RNA virus it was shown that budding from the plasma membranes depends on host functions involved in protein sorting into late endosomal multivesicular bodies (MVBs) [157]. Inhibition of different MVB proteins by coexpression of dominant-negative mutants of AIP1/ALIX, and VPS4B revealed that MVB functions are required for efficient budding and release of enveloped HBV virions. Moreover, HBV virions and subviral particles are all released by distinct pathways with separate host factor requirements [136].

## References

[1] Locarnini S (2004). Molecular virology of hepatitis B virus. Semin Liver Dis.

[2] Lupberger J, Hildt E (2007). Hepatitis B virus-induced oncogenesis. World J Gastroenterol.

[3] Cougot D, Neuveut C, Buendia MA (2005). HBV induced carcinogenesis. J Clin Virol.

[4] Ganem D, Schneider RJ, Knipe DM, Howley PM (2001). Hepadnaviridae: the viruses and their replication. Fields Virology.

[5] Gerlich WH, Heermann KH, Lu X (1992). Functions of hepatitis B surface proteins. Arch Virol Suppl.

[6] Lu X, Mehta A, Dwek R, Butters T, Block T (1995). Evidence that N-linked glycosylation is necessary for hepatitis B virus secretion. Virology.

[7] Schmitt S, Glebe D, Tolle TK, Lochnit G, Linder D, Geyer R, Gerlich WH (2004). Structure of pre-S2 N- and O-linked glycans in surface proteins from different genotypes of hepatitis B virus. J Gen Virol.

[8] Eble BE, Lingappa VR, Ganem D (1990). The N-terminal (pre-S2) domain of a hepatitis B virus surface glycoprotein is translocated across membranes by downstream signal sequences. J Virol.

[9] Eble BE, MacRae DR, Lingappa VR, Ganem D (1987). Multiple topogenic sequences determine the transmembrane orientation of the hepatitis B surface antigen. Mol Cell Biol.

[10] Short JM, Chen S, Roseman AM, Butler G, Crowther RA (2009). Structure of Hepatitis B Surface Antigen from Subviral Tubes Determined by Electron Cryomicroscopy. J Mol Biol.

[11] Bruss V, Ganem D (1991). The role of envelope proteins in hepatitis B virus assembly. Proc Natl Acad Sci U S A.

[12] Dryden KA, Wieland SF, Whitten-Bauer C, Gerin JL, Chisari FV, Yeager M (2006). Native hepatitis B virions and capsids visualized by electron cryomicroscopy. Mol Cell.

[13] Seitz S, Urban S, Antoni C, Bottcher B (2007). Cryo-electron microscopy of hepatitis B virions reveals variability in envelope capsid interactions. EMBO J.

[14] Bruss V, Lu X, Thomssen R, Gerlich WH (1994). Post-translational alterations in transmembrane topology of the hepatitis B virus large envelope protein. EMBO J.

[15] Ostapchuk P, Hearing P, Ganem D (1994). A dramatic shift in the transmembrane topology of a viral envelope glycoprotein accompanies hepatitis B viral morphogenesis. EMBO J.

[16] Prange R, Streeck RE (1995). Novel transmembrane topology of the hepatitis B virus envelope proteins. EMBO J.

[17] Hildt E, Urban S, Hofschneider PH (1995). Characterization of essential domains for the functionality of the MHBst transcriptional activator and identification of a minimal MHBst activator. Oncogene.

[18] Bruss V, Hagelstein J, Gerhardt E, Galle PR (1996). Myristylation of the large surface protein is required for hepatitis B virus in vitro infectivity. Virology.

[19] Gripon P, Le Seyec J, Rumin S, Guguen-Guillouzo C (1995). Myristylation of the hepatitis B virus large surface protein is essential for viral infectivity. Virology.

[20] Rapoport TA, Matlack KE, Plath K, Misselwitz B, Staeck O (1999). Posttranslational protein translocation across the membrane of the endoplasmic reticulum. Biol Chem.

[21] Awe K, Lambert C, Prange R (2008). Mammalian BiP controls posttranslational ER translocation of the hepatitis B virus large envelope protein. FEBS Lett.

[22] Lambert C, Prange R (2003). Chaperone action in the posttranslational topological reorientation of the hepatitis B virus large envelope protein: Implications for translocational regulation. Proc Natl Acad Sci U S A.

[23] Matlack KE, Misselwitz B, Plath K, Rapoport TA (1999). BiP acts as a molecular ratchet during posttranslational transport of prepro-alpha factor across the ER membrane. Cell.

[24] Bruss V (1997). A short linear sequence in the pre-S domain of the large hepatitis B virus envelope protein required for virion formation. J Virol.

[25] Hildt E, Saher G, Bruss V, Hofschneider PH (1996). The hepatitis B virus large surface protein (LHBs) is a transcriptional activator. Virology.

[26] Hildt E, Urban S, Eckerskorn C, Hofschneider PH (1996). Isolation of highly purified, functional carboxy-terminally truncated hepatitis B virus middle surface protein activators from eucaryotic expression systems. Hepatology.

[27] Hildt E, Urban S, Lauer U, Hofschneider PH, Kekule AS (1993). ER-localization and functional expression of the HBV transactivator MHBst. Oncogene.

[28] Hildt E, Munz B, Saher G, Reifenberg K, Hofschneider PH (2002). The PreS2 activator MHBs(t) of hepatitis B virus activates c-raf-1/Erk2 signaling in transgenic mice. EMBO J.

[29] Stockl L, Berting A, Malkowski B, Foerste R, Hofschneider PH, Hildt E (2003). Integrity of c-Raf-1/MEK signal transduction cascade is essential for hepatitis B virus gene expression. Oncogene.

[30] Hafner A, Brandenburg B, Hildt E (2003). Reconstitution of gene expression from a regulatory-protein-deficient hepatitis B virus genome by cell-permeable HBx protein. EMBO Rep.

[31] Huovila AP, Eder AM, Fuller SD (1992). Hepatitis B surface antigen assembles in a post-ER, pre-Golgi compartment. J Cell Biol.

[32] Gilbert RJ, Beales L, Blond D, Simon MN, Lin BY, Chisari FV, Stuart DI, Rowlands DJ (2005). Hepatitis B small surface antigen particles are octahedral. Proc Natl Acad Sci U S A.

[33] Bruns M, Miska S, Chassot S, Will H (1998). Enhancement of hepatitis B virus infection by noninfectious subviral particles. J Virol.

[34] Patient R, Hourioux C, Sizaret PY, Trassard S, Sureau C, Roingeard P (2007). Hepatitis B virus subviral envelope particle morphogenesis and intracellular trafficking. J Virol.

[35] Chain BM, Myers R (2005). Variability and conservation in hepatitis B virus core protein. BMC Microbiol.

[36] Gallina A, Bonelli F, Zentilin L, Rindi G, Muttini M, Milanesi G (1989). A recombinant hepatitis B core antigen polypeptide with the protamine-like domain deleted self-assembles into capsid particles but fails to bind nucleic acids. J Virol.

[37] Zlotnick A, Cheng N, Stahl SJ, Conway JF, Steven AC, Wingfield PT (1997). Localization of the C terminus of the assembly domain of hepatitis B virus capsid protein: implications for morphogenesis and organization of encapsidated RNA. Proc Natl Acad Sci U S A.

[38] Zlotnick A, Cheng N, Conway JF, Booy FP, Steven AC, Stahl SJ, Wingfield PT (1996). Dimorphism of hepatitis B virus capsids is strongly influenced by the C-terminus of the capsid protein. Biochemistry.

[39] Nassal M (1992). The arginine-rich domain of the hepatitis B virus core protein is required for pregenome encapsidation and productive viral positive-strand DNA synthesis but not for virus assembly. J Virol.

[40] Nassal M, Rieger A (1993). An intramolecular disulfide bridge between Cys-7 and Cys61 determines the structure of the secretory core gene product (e antigen) of hepatitis B virus. J Virol.

[41] Zhou S, Standring DN (1992). Hepatitis B virus capsid particles are assembled from core-protein dimer precursors. Proc Natl Acad Sci U S A.

[42] Ceres P, Zlotnick A (2002). Weak protein-protein interactions are sufficient to drive assembly of hepatitis B virus capsids. Biochemistry.

[43] Zlotnick A, Johnson JM, Wingfield PW, Stahl SJ, Endres D (1999). A theoretical model successfully identifies features of hepatitis B virus capsid assembly. Biochemistry.

[44] Bartenschlager R, Schaller H (1992). Hepadnaviral assembly is initiated by polymerase binding to the encapsidation signal in the viral RNA genome. EMBO J.

[45] Beck J, Nassal M (2003). Efficient Hsp90-independent in vitro activation by Hsc70 and Hsp40 of duck hepatitis B virus reverse transcriptase, an assumed Hsp90 client protein. J Biol Chem.

[46] Hirsch RC, Lavine JE, Chang LJ, Varmus HE, Ganem D (1990). Polymerase gene products of hepatitis B viruses are required for genomic RNA packaging as wel as for reverse transcription. Nature.

[47] Junker-Niepmann M, Bartenschlager R, Schaller H (1990). A short cis-acting sequence is required for hepatitis B virus pregenome encapsidation and sufficient for packaging of foreign RNA. EMBO J.

[48] Lott L, Beames B, Notvall L, Lanford RE (2000). Interaction between hepatitis B virus core protein and reverse transcriptase. J Virol.

[49] Stahl M, Retzlaff M, Nassal M, Beck J (2007). Chaperone activation of the hepadnaviral reverse transcriptase for template RNA binding is established by the Hsp70 and stimulated by the Hsp90 system. Nucleic Acids Res.

[50] Kann M, Thomssen R, Kochel HG, Gerlich WH (1993). Characterization of the endogenous protein kinase activity of the hepatitis B virus. Arch Virol Suppl.

[51] Kang H, Yu J, Jung G (2008). Phosphorylation of hepatitis B virus core C-terminally truncated protein (Cp149) by PKC increases capsid assembly and stability. Biochem J.

[52] Crowther RA, Kiselev NA, Bottcher B, Berriman JA, Borisova GP, Ose V, Pumpens P (1994). Three-dimensional structure of hepatitis B virus core particles determined by electron cryomicroscopy. Cell.

[53] Bottcher B, Wynne SA, Crowther RA (1997). Determination of the fold of the core protein of hepatitis B virus by electron cryomicroscopy. Nature.

[54] Conway JF, Cheng N, Zlotnick A, Wingfield PT, Stahl SJ, Steven AC (1997). Visualization of a 4-helix bundle in the hepatitis B virus capsid by cryo-electron microscopy. Nature.

[55] Wynne SA, Crowther RA, Leslie AG (1999). The crystal structure of the human hepatitis B virus capsid. Mol Cell.

[56] Bottcher B, Vogel M, Ploss M, Nassal M (2006). High plasticity of the hepatitis B virus capsid revealed by conformational stress. J Mol Biol.

[57] Perlman DH, Berg EA, O'Connor PB, Costello CE, Hu J (2005). Reverse transcription-associated dephosphorylation of hepadnavirus nucleocapsids. Proc Natl Acad Sci U S A.

[58] Roseman AM, Berriman JA, Wynne SA, Butler PJ, Crowther RA (2005). A structural model for maturation of the hepatitis B virus core. Proc Natl Acad Sci U S A.

[59] Beterams G, Bottcher B, Nassal M (2000). Packaging of up to 240 subunits of a 17 kDa nuclease into the interior of recombinant hepatitis B virus capsids. FEBS Lett.

[60] Kratz PA, Bottcher B, Nassal M (1999). Native display of complete foreign protein domains on the surface of hepatitis B virus capsids. Proc Natl Acad Sci U S A.

[61] Uetrecht C, Versluis C, Watts NR, Roos WH, Wuite GJ, Wingfield PT, Steven AC, Heck AJ (2008). High-resolution mass spectrometry of viral assemblies: molecular composition and stability of dimorphic hepatitis B virus capsids. Proc Natl Acad Sci U S A.

[62] Melegari M, Wolf SK, Schneider RJ (2005). Hepatitis B virus DNA replication is coordinated by core protein serine phosphorylation and HBx expression. J Virol.

[63] Deres K, Schroder CH, Paessens A, Goldmann S, Hacker HJ, Weber O, Kramer T, Niewohner U, Pleiss U, Stoltefuss J, Graef E, Koletzki D, Masantschek RN, Reimann A, Jaeger R, Gross R, Beckermann B, Schlemmer KH, Haebich D, Rubsamen-Waigmann H (2003). Inhibition of hepatitis B virus replication by drug-induced depletion of nucleocapsids. Science.

[64] Stray SJ, Zlotnick A (2006). BAY 41-4109 has multiple effects on Hepatitis B virus capsid assembly. J Mol Recognit.

[65] Bourne C, Lee S, Venkataiah B, Lee A, Korba B, Finn MG, Zlotnick A (2008). Small-molecule effectors of hepatitis B virus capsid assembly give insight into virus life cycle. J Virol.

[66] Stray SJ, Bourne CR, Punna S, Lewis WG, Finn MG, Zlotnick A (2005). A heteroaryldihydropyrimidine activates and can misdirect hepatitis B virus capsid assembly. Proc Natl Acad Sci U S A.

[67] Marsh M, Helenius A (2006). Virus entry: open sesame. Cell.

[68] Gripon P, Diot C, Theze N, Fourel I, Loreal O, Brechot C, Guguen-Guillouzo C (1988). Hepatitis B virus infection of adult human hepatocytes cultured in the presence of dimethyl sulfoxide. J Virol.

[69] Rijntjes PJ, Moshage HJ, Yap SH (1988). In vitro infection of primary cultures of cryopreserved adult human hepatocytes with hepatitis B virus. Virus Res.

[70] Galle PR, Hagelstein J, Kommerell B, Volkmann M, Schranz P, Zentgraf H (1994). In vitro experimental infection of primary human hepatocytes with hepatitis B virus. Gastroenterology.

[71] Gripon P, Diot C, Guguen-Guillouzo C (1993). Reproducible high level infection of cultured adult human hepatocytes by hepatitis B virus: effect of polyethylene glycol on adsorption and penetration. Virology.

[72] Walter E, Keist R, Niederost B, Pult I, Blum HE (1996). Hepatitis B virus infection of tupaia hepatocytes in vitro and in vivo. Hepatology.

[73] Kock J, Nassal M, MacNelly S, Baumert TF, Blum HE, von Weizsacker F (2001). Efficient infection of primary tupaia hepatocytes with purified human and woolly monkey hepatitis B virus. J Virol.

[74] von Weizsacker F, Kock J, MacNelly S, Ren S, Blum HE, Nassal M (2004). The tupaia model for the study of hepatitis B virus: direct infection and HBV genome transduction of primary tupaia hepatocytes. Methods Mol Med.

[75] Neurath AR, Kent SB, Strick N, Parker K (1986). Identification and chemical synthesis of a host cell receptor binding site on hepatitis B virus. Cell.

[76] Paran N, Geiger B, Shaul Y (2001). HBV infection of cell culture: evidence for multivalent and cooperative attachment. EMBO J.

[77] Treichel U, Meyer zum Buschenfelde KH, Dienes HP, Gerken G (1997). Receptor-mediated entry of hepatitis B virus particles into liver cells. Arch Virol.

[78] Treichel U, Schreiter T, Meyer zum Buschenfelde KH, Stockert RJ (1995). High-yield purification and characterization of human asialoglycoprotein receptor. Protein Expr Purif.

[79] Bchini R, Capel F, Dauguet C, Dubanchet S, Petit MA (1990). In vitro infection of human hepatoma (HepG2) cells with hepatitis B virus. J Virol.

[80] Gripon P, Rumin S, Urban S, Le Seyec J, Glaise D, Cannie I, Guyomard C, Lucas J, Trepo C, Guguen-Guillouzo C (2002). Infection of a human hepatoma cell line by hepatitis B virus. Proc Natl Acad Sci U S A.

[81] Nassal M (2008). Hepatitis B viruses: reverse transcription a different way. Virus Res.

[82] Singh M, Dicaire A, Wakil AE, Luscombe C, Sacks SL (2004). Quantitation of hepatitis B virus (HBV) covalently closed circular DNA (cccDNA) in the liver of HBV-infected patients by LightCycler real-time PCR. J Virol Methods.

[83] Lucifora J, Durantel D, Belloni L, Barraud L, Villet S, Vincent IE, Margeridon-Thermet S, Hantz O, Kay A, Levrero M, Zoulim F (2008). Initiation of hepatitis B virus genome replication and production of infectious virus following delivery in HepG2 cells by novel recombinant baculovirus vector. J Gen Virol.

[84] Lupberger J, Mund A, Kock J, Hildt E (2006). Cultivation of HepG2.2.15 on Cytodex-3: higher yield of hepatitis B virus and less subviral particles compared to conventional culture methods. J Hepatol.

[85] Offensperger WB, Offensperger S, Walter E, Blum HE, Gerok W (1991). Sulfated polyanions do not inhibit duck hepatitis B virus infection. Antimicrob Agents Chemother.

[86] Leistner CM, Gruen-Bernhard S, Glebe D (2008). Role of glycosaminoglycans for binding and infection of hepatitis B virus. Cell Microbiol.

[87] Schulze A, Gripon P, Urban S (2007). Hepatitis B virus infection initiates with a large surface protein-dependent binding to heparan sulfate proteoglycans. Hepatology.

[88] Glebe D, Urban S (2007). Viral and cellular determinants involved in hepadnaviral entry. World J Gastroenterol.

[89] Le Seyec J, Chouteau P, Cannie I, Guguen-Guillouzo C, Gripon P (1998). Role of the pre-S2 domain of the large envelope protein in hepatitis B virus assembly and infectivity. J Virol.

[90] De Falco S, Ruvo M, Verdoliva A, Scarallo A, Raimondo D, Raucci A, Fassina G (2001). N-terminal myristylation of HBV preS1 domain enhances receptor recognition. J Pept Res.

[91] Engelke M, Mills K, Seitz S, Simon P, Gripon P, Schnolzer M, Urban S (2006). Characterization of a hepatitis B and hepatitis delta virus receptor binding site. Hepatology.

[92] Glebe D, Urban S, Knoop EV, Cag N, Krass P, Grun S, Bulavaite A, Sasnauskas K, Gerlich WH (2005). Mapping of the hepatitis B virus attachment site by use of infection-inhibiting preS1 lipopeptides and tupaia hepatocytes. Gastroenterology.

[93] Gripon P, Cannie I, Urban S (2005). Efficient inhibition of hepatitis B virus infection by acylated peptides derived from the large viral surface protein. J Virol.

[94] Urban S, Gripon P (2002). Inhibition of duck hepatitis B virus infection by a myristoylated pre-S peptide of the large viral surface protein. J Virol.

[95] Petersen J, Dandri M, Mier W, Lutgehetmann M, Volz T, von Weizsacker F, Haberkorn U, Fischer L, Pollok JM, Erbes B, Seitz S, Urban S (2008). Prevention of hepatitis B virus infection in vivo by entry inhibitors derived from the large envelope protein. Nat Biotechnol.

[96] Glebe D, Aliakbari M, Krass P, Knoop EV, Valerius KP, Gerlich WH (2003). Pre-s1 antigen-dependent infection of Tupaia hepatocyte cultures with human hepatitis B virus. J Virol.

[97] Offensperger WB, Offensperger S, Walter E, Blum HE, Gerok W (1991). Inhibition of duck hepatitis B virus infection by lysosomotropic agents. Virology.

[98] Offensperger WB, Offensperger S, Walter E, Blum HE, Gerok W (1993). Suramin prevents duck hepatitis B virus infection in vivo. Antimicrob Agents Chemother.

[99] Stoeckl L, Funk A, Kopitzki A, Brandenburg B, Oess S, Will H, Sirma H, Hildt E (2006). Identification of a structural motif crucial for infectivity of hepatitis B viruses. Proc Natl Acad Sci U S A.

[100] Chojnacki J, Anderson DA, Grgacic EV (2005). A hydrophobic domain in the large envelope protein is essential for fusion of duck hepatitis B virus at the late endosome. J Virol.

[101] Berting A, Fischer C, Schaefer S, Garten W, Klenk HD, Gerlich WH (2000). Hemifusion activity of a chimeric influenza virus hemagglutinin with a putative fusion peptide from hepatitis B virus. Virus Res.

[102] Epand RM (2003). Fusion peptides and the mechanism of viral fusion. Biochim Biophys Acta.

[103] Nunez E, Yelamos B, Delgado C, Gomez-Gutierrez J, Peterson DL, Gavilanes F (2009). Interaction of preS domains of hepatitis B virus with phospholipid vesicles. Biochim Biophys Acta.

[104] Rodriguez-Crespo I, Nunez E, Yelamos B, Gomez-Gutierrez J, Albar JP, Peterson DL, Gavilanes F (1999). Fusogenic activity of hepadnavirus peptides corresponding to sequences downstream of the putative cleavage site. Virology.

[105] Grgacic EV, Schaller H (2000). A metastable form of the large envelope protein of duck hepatitis B virus: low-pH release results in a transition to a hydrophobic, potentially fusogenic conformation. J Virol.

[106] Oess S, Hildt E (2000). Novel cell permeable motif derived from the PreS2-domain of hepatitis-B virus surface antigens. Gene Ther.

[107] Bleifuss E, Kammertoens T, Hutloff A, Quarcoo D, Dorner M, Straub P, Uckert W, Hildt E (2006). The translocation motif of hepatitis B virus improves protein vaccination. Cell Mol Life Sci.

[108] Brandenburg B, Stockl L, Gutzeit C, Roos M, Lupberger J, Schwartlander R, Gelderblom H, Sauer IM, Hofschneider PH, Hildt E (2005). A novel system for efficient gene transfer into primary human hepatocytes via cell-permeable hepatitis B virus-like particle. Hepatology.

[109] Saher G, Hildt E (1999). Activation of c-Raf-1 kinase signal transduction pathway in alpha(7) integrin-deficient mice. J Biol Chem.

[110] Blanchet M, Sureau C (2007). Infectivity determinants of the hepatitis B virus pre-S domain are confined to the N-terminal 75 amino acid residues. J Virol.

[111] Gudima S, Meier A, Dunbrack R, Taylor J, Bruss V (2007). Two potentially important elements of the hepatitis B virus large envelope protein are dispensable for the infectivity of hepatitis delta virus. J Virol.

[112] Lepere C, Regeard M, Le Seyec J, Gripon P (2007). The translocation motif of hepatitis B virus envelope proteins is dispensable for infectivity. J Virol.

[113] Gudima S, He Y, Chai N, Bruss V, Urban S, Mason W, Taylor J (2008). Primary human hepatocytes are susceptible to infection by hepatitis delta virus assembled with envelope proteins of woodchuck hepatitis virus. J Virol.

[114] Rabe B, Glebe D, Kann M (2006). Lipid-mediated introduction of hepatitis B virus capsids into nonsusceptible cells allows highly efficient replication and facilitates the study of early infection events. J Virol.

[115] Dohner K, Sodeik B (2005). The role of the cytoskeleton during viral infection. Curr Top Microbiol Immunol.

[116] Sodeik B (2000). Mechanisms of viral transport in the cytoplasm. Trends Microbiol.

[117] Huang CJ, Chen YH, Ting LP (2000). Hepatitis B virus core protein interacts with the C-terminal region of actin-binding protein. J Biomed Sci.

[118] Kann M, Bischof A, Gerlich WH (1997). In vitro model for the nuclear transport of the hepadnavirus genome. J Virol.

[119] Kann M, Sodeik B, Vlachou A, Gerlich WH, Helenius A (1999). Phosphorylation-dependent binding of hepatitis B virus core particles to the nuclear pore complex. J Cell Biol.

[120] Rabe B, Vlachou A, Pante N, Helenius A, Kann M (2003). Nuclear import of hepatitis B virus capsids and release of the viral genome. Proc Natl Acad Sci U S A.

[121] Pante N, Kann M (2002). Nuclear pore complex is able to transport macromolecules with diameters of about 39 nm. Mol Biol Cell.

[122] Hillemann A, Brandenburg B, Schmidt U, Roos M, Smirnow I, Lemken ML, Lauer UM, Hildt E (2005). Protein transduction with bacterial cytosine deaminase fused to the TLM intercellular transport motif induces profound chemosensitivity to 5-fluorocytosine in human hepatoma cells. J Hepatol.

[123] Conway JF, Watts NR, Belnap DM, Cheng N, Stahl SJ, Wingfield PT, Steven AC (2003). Characterization of a conformational epitope on hepatitis B virus core antigen and quasiequivalent variations in antibody binding. J Virol.

[124] Lupberger J, Luckow A, Pairan A, Schmidt M, Schaedler S, Hildt E (2009).

[125] Jun-Bin S, Zhi C, Wei-Qin N, Jun F (2003). A quantitative method to detect HBV cccDNA by chimeric primer and real-time polymerase chain reaction. J Virol Methods.

[126] Sun D, Nassal M (2006). Stable HepG2- and Huh7-based human hepatoma cell lines for efficient regulated expression of infectious hepatitis B virus. J Hepatol.

[127] Gao W, Hu J (2007). Formation of hepatitis B virus covalently closed circular DNA: removal of genome-linked protein. J Virol.

[128] Guo H, Jiang D, Zhou T, Cuconati A, Block TM, Guo JT (2007). Characterization of the intracellular deproteinized relaxed circular DNA of hepatitis B virus: an intermediate of covalently closed circular DNA formation. J Virol.

[129] Wang H, Kim S, Ryu WS (2009). DDX3 DEAD-Box RNA helicase inhibits hepatitis B virus reverse transcription by incorporation into nucleocapsids. J Virol.

[130] Seeger C, Mason WS (2000). Hepatitis B virus biology. Microbiol Mol Biol Rev.

[131] Lee GH, Wasser S, Lim SG (2008). Hepatitis B pregenomic RNA splicing--the products, the regulatory mechanisms and its biological significance. Virus Res.

[132] Kock J, Nassal M, Deres K, Blum HE, von Weizsacker F (2004). Hepatitis B virus nucleocapsids formed by carboxy-terminally mutated core proteins contain spliced viral genomes but lack full-size DNA. J Virol.

[133] Le Pogam S, Chua PK, Newman M, Shih C (2005). Exposure of RNA templates and encapsidation of spliced viral RNA are influenced by the arginine-rich domain of human hepatitis B virus core antigen (HBcAg 165–173). J Virol.

[134] Newman M, Suk FM, Cajimat M, Chua PK, Shih C (2003). Stability and morphology comparisons of self-assembled virus-like particles from wild-type and mutant human hepatitis B virus capsid proteins. J Virol.

[135] Lambert C, Doring T, Prange R (2007). Hepatitis B virus maturation is sensitive to functional inhibition of ESCRT-III, Vps4, and gamma 2-adaptin. J Virol.

[136] Watanabe T, Sorensen EM, Naito A, Schott M, Kim S, Ahlquist P (2007). Involvement of host cellular multivesicular body functions in hepatitis B virus budding. Proc Natl Acad Sci U S A.

[137] Bruss V (2007). Hepatitis B virus morphogenesis. World J Gastroenterol.

[138] Zhang YY, Zhang BH, Theele D, Litwin S, Toll E, Summers J (2003). Single-cell analysis of covalently closed circular DNA copy numbers in a hepadnavirus-infected liver. Proc Natl Acad Sci U S A.

[139] Gerelsaikhan T, Tavis JE, Bruss V (1996). Hepatitis B virus nucleocapsid envelopment does not occur without genomic DNA synthesis. J Virol.

[140] Perlman D, Hu J (2003). Duck hepatitis B virus virion secretion requires a double-stranded DNA genome. J Virol.

[141] Wei Y, Tavis JE, Ganem D (1996). Relationship between viral DNA synthesis and virion envelopment in hepatitis B viruses. J Virol.

[142] Liao W, Ou JH (1995). Phosphorylation and nuclear localization of the hepatitis B virus core protein: significance of serine in the three repeated SPRRR motifs. J Virol.

[143] Daub H, Blencke S, Habenberger P, Kurtenbach A, Dennenmoser J, Wissing J, Ullrich A, Cotten M (2002). Identification of SRPK1 and SRPK2 as the major cellular protein kinases phosphorylating hepatitis B virus core protein. J Virol.

[144] Basagoudanavar SH, Perlman DH, Hu J (2007). Regulation of hepadnavirus reverse transcription by dynamic nucleocapsid phosphorylation. J Virol.

[145] Bruss V (2004). Envelopment of the hepatitis B virus nucleocapsid. Virus Res.

[146] Ponsel D, Bruss V (2003). Mapping of amino acid side chains on the surface of hepatitis B virus capsids required for envelopment and virion formation. J Virol.

[147] Fernholz D, Stemler M, Brunetto M, Bonino F, Will H (1991). Replicating and virion secreting hepatitis B mutant virus unable to produce preS2 protein. J Hepatol.

[148] Ueda K, Tsurimoto T, Matsubara K (1991). Three envelope proteins of hepatitis B virus: large S, middle S, and major S proteins needed for the formation of Dane particles. J Virol.

[149] Bruss V, Vieluf K (1995). Functions of the internal pre-S domain of the large surface protein in hepatitis B virus particle morphogenesis. J Virol.

[150] Summers J, Smith PM, Horwich AL (1990). Hepadnavirus envelope proteins regulate covalently closed circular DNA amplification. J Virol.

[151] Summers J, Smith PM, Huang MJ, Yu MS (1991). Morphogenetic and regulatory effects of mutations in the envelope proteins of an avian hepadnavirus. J Virol.

[152] Lenhoff RJ, Summers J (1994). Coordinate regulation of replication and virus assembly by the large envelope protein of an avian hepadnavirus. J Virol.

[153] Poisson F, Severac A, Hourioux C, Goudeau A, Roingeard P (1997). Both pre-S1 and S domains of hepatitis B virus envelope proteins interact with the core particle. Virology.

[154] Loffler-Mary H, Dumortier J, Klentsch-Zimmer C, Prange R (2000). Hepatitis B virus assembly is sensitive to changes in the cytosolic S loop of the envelope proteins. Virology.

[155] Bottcher B, Tsuji N, Takahashi H, Dyson MR, Zhao S, Crowther RA, Murray K (1998). Peptides that block hepatitis B virus assembly: analysis by cryomicroscopy, mutagenesis and transfection. EMBO J.

[156] Mabit H, Schaller H (2000). Intracellular hepadnavirus nucleocapsids are selected for secretion by envelope protein-independent membrane binding. J Virol.

[157] Babst M (2005). A protein's final ESCRT. Traffic.

